# Blue light–tuned selective autophagy: CRY1 intercepts ATG8 to protect HY5

**DOI:** 10.1093/plcell/koaf198

**Published:** 2025-08-11

**Authors:** Jiajun Wang

**Affiliations:** Assistant Features Editor, The Plant Cell, American Society of Plant Biologists; School of Life Sciences, Xiamen Key Laboratory of Plant Genetics, Xiamen University, Xiamen 361102, China

Sunlight not only provides energy for plant photosynthesis but also serves as a signal regulating various aspects of plant growth and development. Plants perceive different wavelengths, intensities, and directions of light through multiple photoreceptors, enabling them to adopt distinct growth and developmental strategies to adjust to diverse light environments (de Wit et al. 2016). The Arabidopsis (*Arabidopsis thaliana*) blue light receptor cryptochrome 1 (CRY1) is localized in both the cytoplasm and nucleus and plays a key role in regulating photomorphogenesis. CRY1 promotes photomorphogenesis by interacting via its C-terminal extension domain with the E3 ubiquitin ligase constitutive photomorphogenic 1 (COP1) and its enhancer suppressor of phyA-105 (SPAs). This interaction inhibits the activity of the COP1-SPA complex, thereby inhibiting the degradation of its substrates such as ELONGATED HYPOCOTYL5 (HY5), a key positive regulator of photomorphogenesis (Qu et al. 2024). However, whether blue light–activated CRY1 promotes HY5 accumulation through other pathways has been unclear. In new work, Lu Jiang, Shilong Zhang, and collaborators (Jiang et al. 2025) uncovered a blue light–dependent mechanism by which CRY1 promotes photomorphogenesis in Arabidopsis by suppressing the selective autophagic degradation of HY5.

Autophagy is a highly conserved degradation mechanism in eukaryotic cells whereby cells encapsulate unnecessary or dysfunctional cytoplasmic components and transport them to vacuoles (in yeast and plants) or lysosomes (in animals) for breakdown and recycling (Zhuang et al. 2024). Autophagy occurs widely throughout various stages of plant growth and development. Under normal conditions, basal autophagy is essential for maintaining cellular homeostasis. Stress conditions such as nutrient starvation, high-intensity light stress, and heat stress induce autophagy, leading to intracellular remodeling and physiological changes to cope with adverse environmental conditions (Yagyu and Yoshimoto 2024). However, whether autophagy is involved in the regulation of photomorphogenesis remains largely unexplored.

Selective autophagy is a form of macroautophagy that involves the formation of double-membrane autophagosomes to encapsulate substrates, requiring the coordinated action of multiple autophagy-related (ATG) proteins at different stages (Zhuang et al. 2024). The lipidation of the ubiquitin-like ATG8 family with phosphatidylethanolamine (PE) is a central step, mediated by a ubiquitin-like conjugation system consisting of ATG7 (E1), ATG3 (E2), and the ATG12–ATG5-ATG16 complex (E3) (Corkery and Wu 2024). Lipidated ATG8 anchors to the autophagosome membrane, contributing to autophagosome formation and expansion, and also serves as a receptor in selective autophagy by recognizing degradation substrates (Corkery and Wu 2024; Zhuang et al. 2024).

Using yeast 2-hybrid screening to identify CRY1 N-terminus (CNT1)-interacting proteins, Jiang et al. found that ATG8i interacts with CNT1. Through a series of in vitro, semi-in vivo, and in vivo techniques, the authors confirmed that the N terminus, but not the C terminus, of CRY1 interacts with all 9 ATG8 isoforms in Arabidopsis (ATG8a–ATG8i), with the interactions between CRY1 and ATG8a/ATG8e being blue light dependent. Further analysis revealed that CRY1 contains ATG8-interacting motifs (AIMs) that interact with the conserved LIR/AIM docking site on ATG8.

To investigate whether the autophagy pathway regulates photomorphogenesis, the authors generated the *atg5 atg7* double mutant and *atg8* nonuple mutant (*atg8n*). Phenotypic analysis revealed that both mutant lines exhibited shorter hypocotyls compared with the wild type, under both dark and blue light, with or without nutrient starvation. This suggests that autophagy pathway promotes skotomorphogenesis in darkness while inhibiting photomorphogenesis under blue light.

Given that autophagy represses photomorphogenesis and HY5 is a central positive regulator of this process, the authors investigated whether ATG8 modulates HY5. Through a series of in vitro, semi-in vivo, and in vivo experiments, they found that ATG8 directly interacts with HY5. In protoplast transformation experiments under nutrient starvation, HY5 was observed to colocalize with ATG8a and ATG8e in autophagosomes, whereas in autophagy-defective mutants (*atg5 atg7* and *atg8n*), HY5 lost its punctate cytoplasmic localization and instead accumulated predominantly in the nucleus. Protein level analysis revealed that both endogenous HY5 and overexpressed HY5-GFP were degraded via nutrient starvation-induced autophagy, a process requiring ATG5/7 and ATG8. To further explore the genetic relationship, the authors generated *atg5 atg7 hy5* triple mutants and *atg8n hy5* decuple mutants. The short hypocotyl phenotype observed in *atg5 atg7* and *atg8n* mutants was largely rescued in these higher-order mutant backgrounds. Notably, the hypocotyls of *atg5 atg7 hy5* and *atg8n hy5* mutants were shorter than those of *hy5* under blue light with nutrient starvation, whereas under the other 3 conditions, they were comparable to *hy5*. These findings suggest that HY5 functions at least partially downstream of ATG5/7 and ATG8 in regulating photomorphogenesis.

The authors also assessed the genetic interaction between CRY1 and the autophagy pathway by generating *cry1 atg5 atg7* triple and *cry1 atg8n* decuple mutants. Hypocotyl lengths of these mutants were shorter than *cry1* but longer than those of *atg5 atg7* and *atg8n*, suggesting that ATG5/7 and ATG8 act partially downstream of CRY1. Given that ATG8 plays a critical role in autophagosome formation, the authors examined whether CRY1 regulates this ATG8-mediated process. Confocal microscopy and nuclear-cytoplasmic fractionation assays revealed that blue light significantly suppressed the formation of YFP-ATG8a and YFP-ATG8e puncta (representing autophagosomes) and promoted nuclear accumulation of ATG8e in darkness with nutrient starvation, with both effects being dependent on CRY1.

Since CRY1 inhibits autophagosome formation, the authors further examined whether CRY1 suppresses HY5 degradation via autophagy. Protoplast transformation assays showed that nutrient starvation-induced HY5 localization to autophagosomes in darkness was inhibited by blue light, and this inhibition depended on CRY1. Moreover, blue light-activated CRY1 significantly suppressed HY5 colocalization with ATG8a and ATG8e in autophagosomes and inhibited HY5 degradation via autophagy. Finally, semi–in vivo pull-down assays and co-immunoprecipitation experiments demonstrated that blue light inhibits the interaction between HY5 and ATG8 via CRY1, and this inhibition requires the AIM motif of CRY1, which mediates its interaction with ATG8.

In summary, this study uncovers a mechanism by which blue light–activated CRY1 inhibits autophagy-mediated degradation of HY5 by blocking its interaction with ATG8 and suppressing autophagosome formation (Figure). This work provides evidence for a functional role of autophagy in photomorphogenesis and reveals a HY5 regulatory pathway that operates independently of the COP1–SPA complex.

**Figure.**
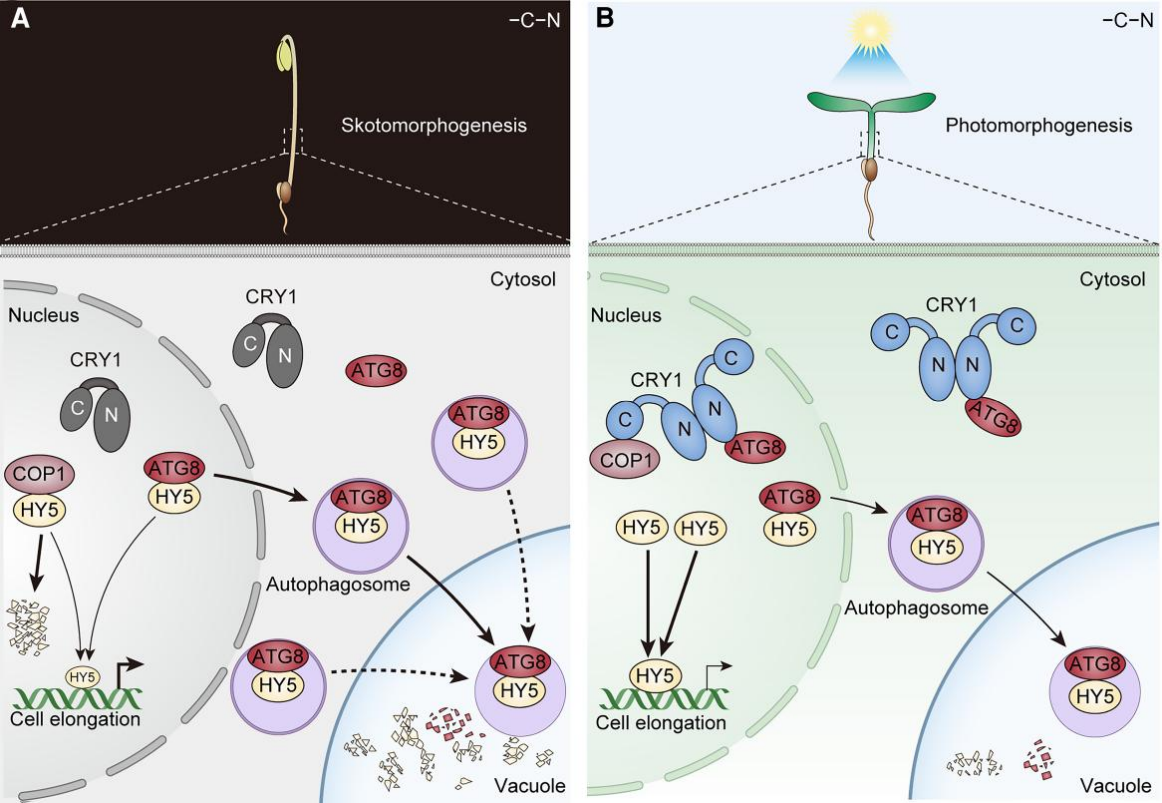
Working model of CRY1-mediated inhibition of HY5 autophagic degradation through ATG8 in response to blue light. **A)** In darkness, CRY1 predominantly exists as a monomer and cannot interact with ATG8. HY5 interacts with ATG8 and is delivered into autophagosomes for selective degradation in the vacuole. Meanwhile, nuclear HY5 is also targeted for ubiquitin-mediated degradation by the COP1-SPA complex. These two mechanisms work together to degrade HY5 and promote skotomorphogenesis. **B)** Upon blue light exposure, CRY1 undergoes oligomerization and becomes activated. Activated CRY1 interacts with ATG8, suppresses autophagosome formation, inhibits the interaction between ATG8 and HY5, and prevents HY5 from being targeted for autophagic degradation, thereby promoting HY5 accumulation and facilitating photomorphogenesis. Reprinted from Jiang et al. (2025), Figure 10.

## Recent related articles in *The Plant Cell*:

- Huang et al. (2023) revealed that the acetyltransferase HOOKLESS1 (HLS1) promotes starvation-induced autophagy in *Arabidopsis* by directly acetylating ATG18a, thereby enhancing its PtdIns(3)P-binding activity and autophagosome formation, independently of HLS1's role in hook development.
- In the review by Otegui et al. (2024), the authors address how selective autophagy mediates vacuolar degradation of various plant organelles, particularly under environmental stress.
- Isono et al. (2024) review the mechanisms of substrate recognition and degradation pathways in plants, focusing on how diverse degron signals direct proteins to specific degradation systems, including the ubiquitin-proteasome system, autophagy, and organellar proteases for proteome remodeling under varying cellular conditions.

## Data Availability

N/A.

## Dive Curated Terms

The following phenotypic, genotypic, and functional terms are of significance to the work described in this paper:

CRY1 Gramene: ostta15g0100
CRY1 Araport: ostta15g0100

HY5 Gramene: At5g11260
HY5 Araport: At5g11260

HYPOCOTYL AmiGo: PO:0020100

COP1 Gramene: AT2G32950
COP1 Araport: AT2G32950

ATG5 Gramene: At5g17290
ATG5 Araport: At5g17290

ATG7 Gramene: At5g45900
ATG7 Araport: At5g45900
